# Molecular profiling of metastatic colorectal tumors using next-generation sequencing: a single-institution experience

**DOI:** 10.18632/oncotarget.15030

**Published:** 2017-02-02

**Authors:** Jun Gong, May Cho, Marvin Sy, Ravi Salgia, Marwan Fakih

**Affiliations:** ^1^ Department of Medical Oncology, City of Hope National Medical Center, Duarte, CA, USA

**Keywords:** metastatic colorectal cancer, comprehensive genomic profiling, next-generation sequencing, FoundationOne, retrospective

## Abstract

**Background:**

Recent molecular characterization of colorectal tumors has identified several molecular alterations of interest that are considered targetable in metastatic colorectal cancer (mCRC).

**Methods:**

We conducted a single-institution, retrospective study based on comprehensive genomic profiling of tumors from 138 patients with mCRC using next-generation sequencing (NGS) via FoundationOne.

**Results:**

Overall, *RAS* mutations were present in 51.4% and *RAF* mutations were seen in 7.2% of mCRC patients. We found a novel *KRAS*^R68S1^ mutation associated with an aggressive phenotype. *RAS* amplifications (1.4% *KRAS* and 0.7% *NRAS*), *MET* amplifications (2.2%), *BRAF*^L597R^alterations (0.7%), *ARAF*^S214F^ alterations (0.7%), and concurrent *RAS*+*RAF* (1.4%), *BRAF*+*RAF1* (0.7%), and rare PTEN-PIK3CA-AKT pathway mutations were identified and predominantly associated with poor prognosis. *ERBB2* (*HER2*) amplified tumors were identified in 5.1% and all arose from the rectosigmoid colon. Three cases (2.2%) were associated with a hypermutated profile that was corroborated with findings of high tumor mutational burden (TMB): 2 cases with MSI-H and 1 case with a *POLE* mutation.

**Conclusions:**

Comprehensive genomic profiling can uncover alterations beyond the well-characterized *RAS*/*RAF* mutations associated with anti-EGFR resistance. *ERBB2* amplified tumors commonly originate from the rectosigmoid colon, are predominantly *RAS*/*BRAF* wild-type, and may predict benefit to HER2-directed therapy. Hypermutant tumors or tumors with high TMB correlate with MSI-H status or *POLE* mutations and may predict a benefit from anti-PD-1 therapy.

## INTRODUCTION

Colorectal cancer (CRC) remains the third leading cause of cancer death in both men and women in the United States with an estimated 134,490 new cases and 49,190 deaths in 2016 [1]. Recent advances in the treatment of metastatic CRC (mCRC) have identified improved outcomes with the addition of epidermal growth factor receptor (EGFR)-targeting agents to conventional combination cytotoxic therapy in patients with extended *RAS* wild-type tumors. In contrast, activating mutations in the *RAS* gene (*KRAS* or *NRAS*, present in approximately 50% of cases of mCRC) and *BRAF* gene (present in about 5% of mCRC patients) have been associated with lack of clinically meaningful benefit or harm when anti-EGFR therapy is employed [2]. The identification of candidates for anti-EGFR therapy through the exclusion of *RAS* and *BRAF* mutations in mCRC serves as a model of selecting optimal therapy based on patient genomic profiles and molecular phenotypes.

Several decades of genomic studies, including the use of more recent next-generation sequencing (NGS), have expedited the search of genetic alterations for potential therapeutic targeting in CRC [3, 4]. Recently, comprehensive molecular characterization of 224 colorectal tumors was performed by The Cancer Genome Atlas (TCGA) Network [5]. Sixteen percent of colorectal tumors were found to be hypermutated and more commonly found in the right colon with 75% of these cases demonstrating expectedly high microsatellite instability (MSI-H). Twenty-four genes were identified to have significant mutations of interest including *APC*, *SMAD4*, *TP53*, *PIK3CA*, and *KRAS* mutations, as expected. Interestingly, mutations, deletions, or amplifications of the *ERRB* gene family were found in 19% of tumors. In sum, this genomic analysis identified several molecular alterations that are considered targetable, including mediators of dysregulated WNT, RAS, and PI3K pathways such as ERRB2, ERRB3, MEK, AKT, MTOR, IGF2, and IGFR.

The recent identification of gene mutations and amplifications of potential significance for therapeutic purposes has led us to investigate the genomic profiles of mCRC patients using NGS (FoundationOne). Here, we describe a single-institution experience in reporting results from comprehensive genomic analysis of tumors from 138 mCRC patients. We aim to characterize genetic alterations present in our study population that have known correlates to prognosis, therapeutic resistance, and potential therapeutic targets in mCRC. In this study, we also report the existence of concurrent gene mutations rarely described in the literature and novel mutations and amplifications that can lead to targeting outside of National Comprehensive Cancer Network (NCCN) standard treatments.

## RESULTS

### Study population

The molecular results from FoundationOne testing of tumors from 138 mCRC patients are summarized in Table 1. The median age of our study group was 56 years (range 27-88) with 59.4% (82) males and 40.6% (56) females. The most common ethnicity was White (85, 61.6%) followed by Asian (29, 21.0%). The most common sites of primary were sigmoid colon (33.3%), rectum (19.6%), and cecum (15.2%). Sixty-eight patients (49.3%) had *KRAS* mutations, 9 patients (6.5%) had *BRAF* mutations, 3 (2.2%) had *NRAS* mutations, 1 (0.7%) had an *ARAF* mutation, 1 (0.7%) had a *RAF-1* mutation, 7 (5.1%) had *ERRB2* amplifications, 25 (18.1%) had *PIK3CA* mutations, 15 (10.9%) had *PTEN* mutations, 4 (2.9%) had *AKT* mutations, and 3 (2.2%) had *MET* amplifications.

**Table 1:** Demographics/patient characteristics

Characteristic (n = 138)	Frequency (%)
Age (at initial diagnosis)	Median (range)
	56 (27-88)
Sex	
Male	82 (59.4%)
Female	56 (40.6%)
Ethnicity	
White	85 (61.6%)
Asian	29 (21.0%)
Black	7 (5.1%)
Other	3 (2.2%)
Unknown	14 (10.1%)
Primary disease site	
Sigmoid colon	46 (33.3%)
Rectal	27 (19.6%)
Cecum	21 (15.2%)
Rectosigmoid	13 (9.4%)
Colon NOS	31 (22.5%)
Stage (at diagnosis)	
II	5 (3.6%)
III	16 (11.6%)
IV	117 (84.8%)
Relapsed disease	
Yes	52 (37.7%)
No	86 (62.3%)
KRAS alterations	68 (49.3%)
G12D	23 (16.7%)
G13D	10 (7.2%)
G12V	8 (5.8%)
G12S	7 (5.1%)
G12C	6 (4.3%)
A146T	3 (2.2%)
G12A	2 (1.4%)
Amplification	2 (1.4%)
Q61H	1 (0.7%)
Q61K	1 (0.7%)
Q61L	1 (0.7%)
K117N	1 (0.7%)
R68S	1 (0.7%)
A146V	1 (0.7%)
A146V^sub	1 (0.7%)
BRAF alterations	9 (6.5%)
V600E	5 (3.6%)
D594G	1 (0.7%)
G466V	1 (0.7%)
G469E	1 (0.7%)
L597R	1 (0.7%)
NRAS alterations	
Q61K	2 (1.4%)
Amplification	1 (0.7%)
ARAF mutation	
S214F	1 (0.7%)
RAF1 mutation	
S257L	1 (0.7%)
ERBB2 alterations	
Amplification	7 (5.1%)
MET alterations	
Amplification	3 (2.2%)
AKT1/AKT2 mutations	
AKT1	3 (2.2%)
AKT2	1 (0.7%)
Total number of clinically significant alterations	Median (range) 5 (1-25)
MSI	
MSS	121 (87.7%)
MSI-L	0 (0.0%)
MSI-H	2 (1.4%)
Unknown/not reported	15 (10.9%)

NOS: Not otherwise specified; MSI: Microsatellite instability; MSS: Microsatellite stable; MSI-L: MSI low; MSI-H: MSI high.

### RAS mutations

**Figure 1:**
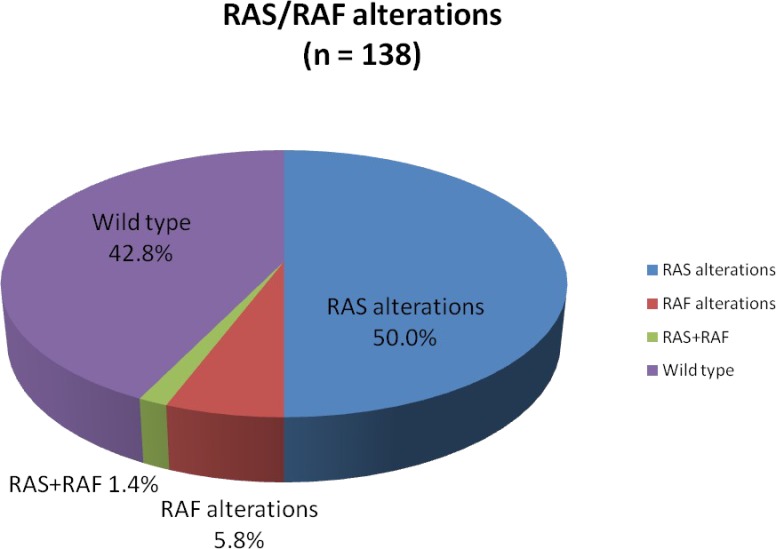
Proportion of RAS, RAF, RAS+RAF mutations, and RAS/RAF wild type status identified by comprehensive genomic profiling RAS+RAF mutations are not included in RAS or RAF percentages.

**Figure 2:**
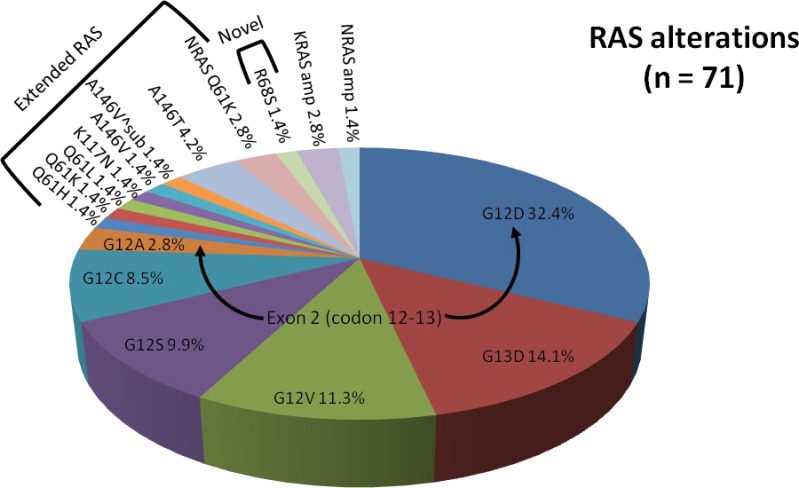
Proportion of RAS alterations identified by comprehensive genomic profiling Arrows denote common mutations of exon 2 (codon 12-13). Brackets denote panel of extended RAS mutations or novel RAS mutation.

Overall, *RAS* mutations were present in 51.4% of our mCRC patients, *RAF* mutations were seen in 7.2%, of which *RAS*+*RAF* concurrent mutations were seen in 1.4%. The remainder (42.8%) were *RAS*/*RAF* wild type (Figure 1). The most common *RAS* mutations were *KRAS* mutations of exon 2 (codons 12 and 13) including G12D (32.4%), G13D (14.1%), G12V (11.3%), G12S (9.9%), G12C (8.5%), and G12A (2.8%, Figure 2). Beyond the well-established point mutations in codons 12 and 13 of exon 2 of *KRAS*, we identified mutations in codon 61 of exon 3 (Q61H, 1.4%; Q61K, 1.4%; Q61L, 1.4%), codon 117 of exon 4 (K117N, 1.4%), and codon 146 of exon 4 (A146V, 1.4%; A146V^sub, 1.4%; A146T, 4.2%). Two mutations (2.8%) in codon 61 (exon 3) of *NRAS* were also detected. Altogether, these non-*KRAS* exon 2 mutations constitute 15.5% of *RAS* mutations.

In our patient population, 2 *KRAS* amplifications (2.8%) and 1 *NRAS* amplification (1.4%) were identified. One patient was a 51-year-old female with *KRAS* amplified rectal cancer with synchronous diffuse metastases (lung and liver). Her best overall response to standard first-line combination chemotherapy (5-fluorouracil (5-FU) and irinotecan or FOLFIRI) plus anti-EGFR therapy (panitumumab) was stable disease (SD) for 6 months. The other patient with *KRAS* amplification was a 51-year-old male diagnosed with right-sided colon cancer and synchronous metastases to the liver and peritoneum who had rapid progression on first- and second-line non-anti-EGFR based therapies. Our 74-year-old male patient with *NRAS* amplification presented with poorly differentiated rectosigmoid adenocarcinoma and synchronous diffuse metastases (liver, mesentery, and bones) and experienced progressive disease (PD) at 2 months on second-line FOLFIRI + cetuximab. Notably, a novel *KRAS*^R68S1^ alteration (Figure 2) was identified in a 41-year-old female (1.4%) with rectal cancer and synchronous metastases to the liver and retroperitoneal and supraclavicular lymph nodes who experienced PD at 2 months on anti-EGFR therapy with second-line irinotecan + cetuximab.

### RAF mutations

**Figure 3:**
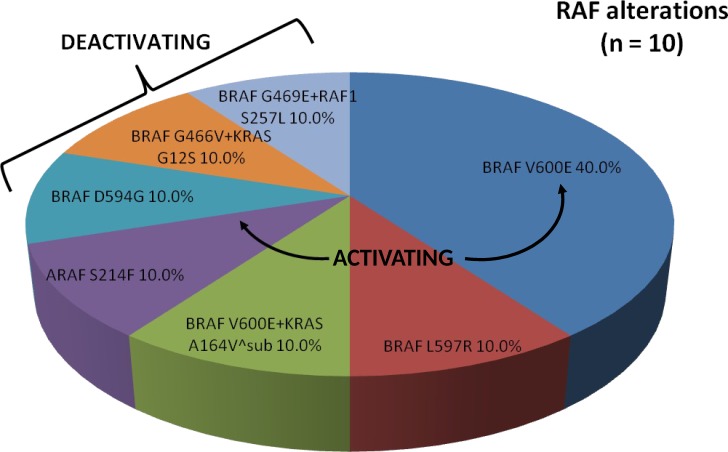
Proportion of RAF alterations identified by comprehensive genomic profiling Arrows denote known activating mutations. Brackets denote known deactivating mutations.

A total of 11 *RAF* mutations (1 concurrent *BRAF*+*RAF1* mutation) were found in 7.2% of our patients (Figure 3). Of these, *BRAF*^V600E^ activating mutations (exon 15) were the most common single mutations present (40.0%). One activating *BRAF*^L597R^alteration (exon 15) was identified (10.0%) in a 56-year-old male with bulky rectal adenocarcinoma with synchronous metastases that progressed through 9 months of first-line anti-EGFR therapy. One activating *ARAF*^S214F^ alteration was also identified (10.0%) in our series of *RAF* mutations. This 60-year-old male patient developed multiple recurrences of rectal adenocarcinoma including, most recently, metastatic disease to the lung treated with neoadjuvant 5-FU, oxaliplatin, and irinotecan (FOLFOXIRI) followed by metastatectomy; he remains in clinical remission. A dual *BRAF*^V600E^+*KRAS*^A164V^sub^alterationwas present (10.0%) in an elderly male (age 72) with poorly differentiated right-sided colon cancer with synchronous metastases on first-line systemic combination therapy without anti-EGFR agents. Here an oncogenic *RAS* alteration was paired with a known activating *BRAF* mutation.

Deactivating mutations in *BRAF*^D594G^(10.0%), *BRAF*^G466V^ concurrent with *KRAS*^G12S^ (10.0%), and *BRAF*^G469E^ concurrent with *RAF1*^S257L^ (10.0%) were also identified. Our patient with a deactivating *BRAF*^D594G^ mutation was a 59-year-old male with moderately-poorly differentiated right-sided colon cancer with diffuse metastases that was refractory to all standard of care chemotherapy, including FOLFIRI + cetuximab, and ultimately died from progressive disease. Interestingly, he was noted to have a concurrent *MET* amplification. The patient with a deactivating *BRAF*^G466V^ mutation concurrent with an activating *KRAS*^G12S^ mutation was a 51-year-old male with right-sided colon cancer with diffuse metastases that progressed with carcinomatosis while on FOLFOX and immediately following salvage debulking surgery with hyperthermic chemotherapy. Notably, he currently has achieved ongoing partial response (PR) on third-line FOLFIRI and bevacizumab (41+ cycles). Our 66-year-old female with dual deactivating *BRAF*^G469E^ and activating *RAF1*^S257L^ mutation presented with a right colon cancer with synchronous metastases to bone, liver, lung, and peritoneum. Her disease was refractory to FOLFOX + bevacizumab and is currently on second-line FOLFIRI + bevacizumab with a clinical benefit.

### ERBB2 amplifications

**Figure 4:**
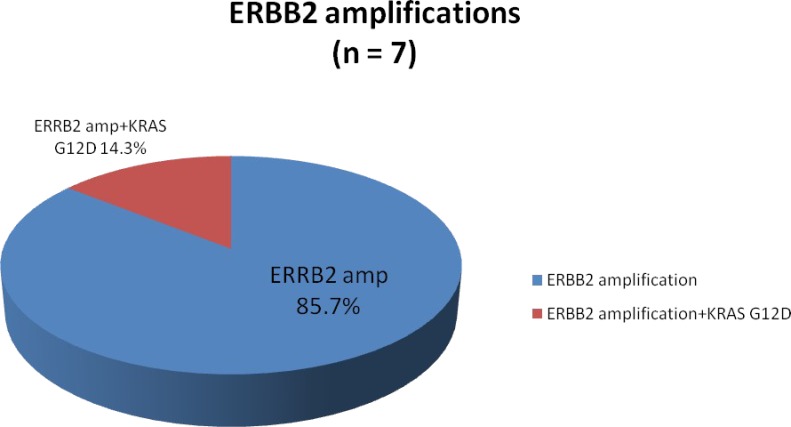
Proportion of ERBB2 amplifications identified by comprehensive genomic profiling

Seven patients (5.1%) were found to have *ERRB2* amplified tumors with one having a concurrent *KRAS*^G12D^ mutation (Figure 4). The majority of these tumors were MSS (87.5%) with *HER2* copy numbers that ranged from 9-190 (Table 2). Notably, all *ERRB2* amplified tumors were located in the rectosigmoid colon as its primary disease site. Four patients with *RAS* wild-type *ERBB2* amplification received anti-EGFR therapy, 3 experienced SD ≥ 4 months (2 first-line and 1 second-line) and 1 (second-line) experienced a PR lasting for 5 months as their best overall response to anti-EGFR therapy. The concurrent *ERRB2* amplified and *KRAS*^G12D^ mutated tumor was found in a 58-year-old male with moderately differentiated rectal adenocarcinoma with synchronous solitary liver metastasis treated with neoadjuvant 5-FU, oxaliplatin (FOLFOX) followed by hepatic resection and resection of primary – he is currently under surveillance and without evidence of disease.

**Table 2:** Subset of ERBB2 amplifications

Copy number	Dual mutations	Age	Race/sex	Primary disease site	Lines of systemic therapy	Best overall response to anti-EGFR	MSI
9		55	Latino/M	Rectum	2	SD (4 mo)	NR
12		88	Middle Eastern/M	Sigmoid	1		MSS
37		50	White/F	Rectum	2		MSS
77	KRAS G12D	58	White/M	Rectum	2		MSS
93		60	White/M	Rectosigmoid	2	PR (5 mo)	MSS
189		48	White/M	Sigmoid	4	SD (4 mo)	MSS
190		71	Asian/M	Sigmoid	2	SD (5 mo)	MSS

EGFR: Epidermal growth factor receptor; MSI: Microsatellite instability; SD: Stable disease; NR: Not reported; MSS: Microsatellite stable; PR: Partial response.

### AKT1/2 mutations

Three patients (2.2%) had *AKT1*^E17K^ mutations while 1 patient (0.7%) had an *AKT2*^E17K^ mutation (Table 3). Of these, a majority had concurrent mutations (75%) and tumors located in the right colon (75%). One *AKT1*^E17K^ mutated tumor was found to have concurrent *BRAF*^V600E^ +*KRAS*^A164V^sub^alterations with phenotype described above. One *AKT1*^E17K^ mutated tumor had concurrent alterations in *KRAS*^A146T^+*PIK3CA*^G106V^ and was found in a 61-year-old male with initial right-sided colon cancer that recurred with metastases to the liver showing moderately differentiated colon adenocarcinoma. His tumor was characterized by aggressive features, including metastatic disease recurrence following a diagnosis of stage I disease, and development of bony metastases within the first year of recurrence. A concurrent *AKT2*^E17K^+*KRAS*^G12C^ altered tumor was found in a 57-year-old female with originally moderately differentiated sigmoid adenocarcinoma that was resected but recurred with metastases to the retroperitoneal lymph nodes currently on first-line FOLFIRI + bevacizumab.

**Table 3:** Subset of AKT1/2 mutations

Variant	Dual mutations*	Age	Race/Sex	Primary disease site	Lines of systemic therapy	MSI
E17K (AKT1)	KRAS A146T	61	Black/M	Right colon, NOS	2	NR
E17K (AKT1)	BRAF V600E, KRAS A146^sub	72	White/M	Ascending colon	1	MSS
E17K (AKT1)		69	White/F	Ascending colon	1	MSS
E17K (AKT2)	KRAS G12C	57	Asian/F	Sigmoid	1	MSS

* For concurrent *RAS* and *BRAF* mutations only, NOS: Not otherwise specified; MSI: Microsatellite instability; NR: Not reported; MSS: Microsatellite stable.

### PIK3CA and PTEN mutations

**Figure 5:**
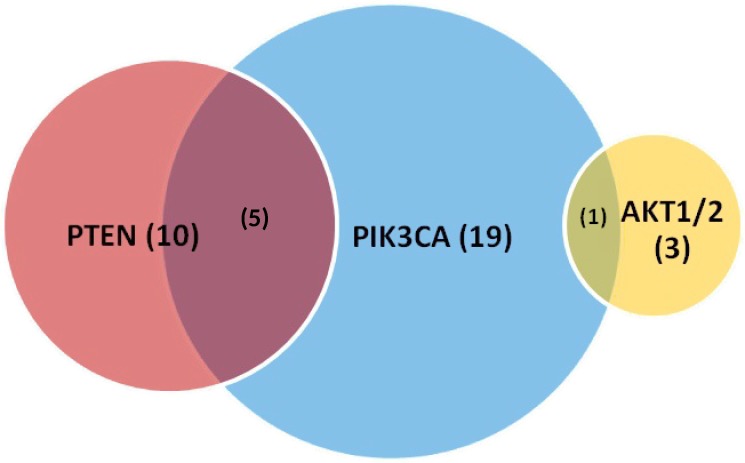
Proportion of PIK3CA, PTEN, AKT1/2 mutations identified by comprehensive genomic profiling with overlap Values in parentheses represent numbers and not percentages.

In total, we identified 25 patients (18.1%) with *PIK3CA* alterations in our cohort (Table 4). The most common primary disease sites included cecum (36.0%), sigmoid colon (12.0%), and rectum (12.0%). Notably, right-sided colon cancers comprised nearly half (48.0%) of tumors with *PIK3CA* alterations. The most commonly identified variants were E545K (24.0%, exon 9), E542K (12.0% exon 9), E110del (8.0%), and Q546K (8.0%). Tumors with *PIK3CA* alterations frequently had concurrent mutations in the RAS-RAF-MAPK signaling pathway. A majority (19 or 76.0%) had concurrent mutations in *KRAS* (G12D 36.0%, G12S 12.0%, G13D 8.0%, and A146T 8.0%). Two patients (8.0%) with *PIK3CA* tumors were found to have concurrent deactivating *BRAF* mutations (G466V and G469E). Notably, these 2 patients had additional alterations in *KRAS*^G12S^ and *RAF1*^S257L^, respectively, with phenotypes described above. We also identified additional alterations in the PTEN-PIK3CA-AKT signaling pathway in our group of *PIK3CA* altered tumors (Figure 5). Five patients (20.0%) with *PIK3CA* altered tumors also had *PTEN* mutations, while 1 patient (4.0%) had a dual *PIK3CA* and *AKT1* mutated tumor. Of note, 1 female patient (age 55) with a dual *PIK3CA* and *PTEN* mutation had a rectal tumor demonstrating MSI-H and developed a solitary liver metastasis that has since been resected and treated with adjuvant FOLFOX – she is currently in remission.

**Table 4:** Subset of PIK3CA alterations

Characteristic (n = 25)	Frequency (%)
Age (at initial diagnosis)	Median (range) 55 (30-81)
Sex	
Male	14 (56.0%)
Female	11 (44.0%)
Ethnicity	
White	17 (68.0%)
Asian	2 (8.0%)
Black	2 (8.0%)
Other/unknown	4 (16.0%)
Primary disease site	
Right colon	12 (48.0%)
Transverse colon	2 (8.0%)
Left colon (includes sigmoid)	8 (32.0%)
Rectum	3 (12.0%)
Variant	
E545K (exon 9)	6 (24.0%)
E542K (exon 9)	3 (12.0%)
E110del	2 (8.0%)
Q546K	2 (8.0%)
C901F	1 (4.0%)
C420R	1 (4.0%)
C420R+E726K	1 (4.0%)
E545G^sub+R108H^sub	1 (4.0%)
G106V	1 (4.0%)
H1047L (exon 20)	1 (4.0%)
H1047Y (exon 20)	1 (4.0%)
N107del	1 (4.0%)
N345K	1 (4.0%)
P104L	1 (4.0%)
P104_V105del	1 (4.0%)
R88Q	1 (4.0%)
Concurrent KRAS mutations	19 (76.0%)
G12D	9 (36.0%)
G12S	3 (12.0%)
G13D	2 (8.0%)
A146T	2 (8.0%)
G12V	1 (4.0%)
G12C	1 (4.0%)
A146V	1 (4.0%)
Concurrent PTEN mutations	5 (20.0%)
H93Y^sub	1 (4.0%)
L316fs*7	1 (4.0%)
L57fs*6+N323fs*2	1 (4.0%)
R130Q+R142W	1 (4.0%)
Splice site 1008_1026+5del24	1 (4.0%)
Concurrent BRAF mutations	
G466V	1 (4.0%)
G469E	1 (4.0%)
Concurrent AKT1 mutation	
E17K	1 (4.0%)
MSI	
MSS	21 (84.0%)
MSI-L	0 (0.0%)
MSI-H	1 (4.0%)
Unknown/Not reported	3 (12.0%)

MSI: Microsatellite instability; MSS: Microsatellite stable; MSI-L: MSI low; MSI-H: MSI high.

### MET amplifications

Three patients (2.2%) in our series had *MET* amplifications (Table 5). Two-thirds of these tumors were MSS, located in the right colon, and associated with concurrent mutations in *RAS* or *RAF* genes. One 67-year-old male was initially diagnosed with right-sided colon cancer (*KRAS*^G13D^+*MET* alterations present) and synchronous liver metastases. His course has been punctuated by recurrent metastases to the liver and lungs despite several systemic and regional therapies. Another right-sided colon cancer was identified with both a deactivating *BRAF*^D594G^ mutation and *MET* amplification with aggressive phenotype described above. A third patient was a 27-year-old male with primary rectal adenocarcinoma that recurred with metastases to the liver and retroperitoneal lymph nodes and refractory to capecitabine + irinotecan + cetuximab. In particular, 2 of 3 patients with *MET* amplications and *RAS*/*BRAF*^V600E^ wild-type tumors were refractory to anti-EGFR-based therapies.

**Table 5:** Subset of MET amplifications

Variant	Dual Mutations*	Age	Race/Sex	Smoker	Primary disease site	Lines of systemic therapy	MSI
MET	BRAF D594G	59	Asian/M	Yes	Right colon, NOS	4	MSS
MET		27	White/M	Yes	Rectum	2	NR
MET	KRAS G13D	67	Asian/M	No	Ascending colon	1	MSS

* For concurrent *RAS* and *BRAF* mutations only, MSI: Microsatellite instability; NOS: Not otherwise specified; NR: Not reported; MSS: Microsatellite stable.

### Hypermutant status

The majority of our 138 patients with mCRC had tumors with <9 clinically significant alterations (121 or 87.7%) as described by FoundationOne reports (Table 6). The majority of these tumors were located in the left colon and all were MSS. Fourteen patients (10.1%) had 9-16 total alterations while only 3 patients (2.2%) were allocated to the highest number of clinically significant alterations category (17-25). Notably, 2 patients with MSI-H tumors were identified in the highest number of alterations group. One 55-year-old female patient was found to have a dual *PIK3CA* and *PTEN* mutated rectal tumor with phenotype described previously. Tumor mutational burden (TMB) from FoundationOne report showed a high TMB of 33 mutations per megabase (Mb). The other was a 47-year-old female with *KRAS*^G12V^ mutated metastatic rectal cancer that has progressed through 3 lines of systemic therapy and currently on anti-PD-1 therapy with pembrolizumab with a clinical response. Again, TMB corroborated her findings of a relatively hypermutated tumor with a TMB of 31 mutations/Mb. The third patient with a hypermutant FoundationOne profile had a MSS tumor with an associated *POLE*^V411L^ mutation. Interestingly, this patient was elderly (age 80), had a right colon tumor, and had a recurrence pattern consistent with locoregional recurrence. This patient demonstrated a TMB of 122 mutations/Mb, which was the highest among the cohort.

**Table 6:** Total number of clinically significant alterations

No. of alterations	Frequency (%)	Age	Sex	Race	Primary disease site	MSI
<9	121 (87.7%)	Median (range) 56 (27-84)	F 48 (39.7%) M 73 (60.3%)	White 74 (61.2%) Asian 25 (20.7%) Black 6 (4.9%) Other 16 (13.2%)	Right colon 28 (23.1%) Transverse colon 5 (4.1%) Left colon (includes sigmoid) 60 (49.6%) Rectum 22 (18.2%) Colon, NOS 6 (5.0%)	MSS 121 (100%)
9-16	14 (10.1%)	Median (range) 60 (39-88)	F 6 (42.9%)M 8 57.1%)	White 8 (57.1%) Asian 4 (28.7%) Black 1 (7.1%) Unknown 1 (7.1%)	Right colon 6 (42.9%) Left colon (includes sigmoid) 5 (35.7%) Rectum 3 (21.4%)	MSS 13 (92.9%) NR 1 (7.1%)
17-25	3 (2.2%)	Mean 59.7	F 2 (66.6%) M 1 (33.3%)	White 3 (100%)	Rectum 2 (66.6%) Right colon 1 (33.3%)	MSI-H 2 (66.6%) MSS 1 (33.3%)

MSI: Microsatellite instability; NOS: Not otherwise specified; MSS: Microsatellite stable; NR: Not reported; MSI-H: MSI high.

## DISCUSSION

Comprehensive molecular characterization of 138 tumors from patients with mCRC was performed via NGS (FoundationOne) in this single-institution retrospective study. Overall, 51.4% and 7.2% of our patients with mCRC were shown to carry *RAS* and *RAF* mutations, respectively, which is concordant with frequencies historically reported in mCRC [2]. The majority of our *RAS* mutations were *KRAS* mutations of exon 2 (codons 12 and 13), which represent those identified in initial phase III trials that predicted lack of benefit from anti-EGFR therapy in mCRC [6, 7]. We also found that 15.5% of all *RAS* mutations in our population comprised a panel of extended *RAS* mutations. This is also consistent with recent data from the PRIME and CRYSTAL clinical trials, where exon 3 and 4 *KRAS* and exons 2, 3, and 4 *NRAS* mutations reflected 14-17% of *RAS* mutations [8, 9]. Identifying these rare *RAS* mutations has major clinical significance, given their association with anti-EGFR resistance [10].

Notably, we identified 2 *KRAS* amplifications and 1 *NRAS* amplification that are extremely rare and poorly characterized. These were found in 3 patients with diffusely metastatic CRC progressive through several lines of systemic therapy including anti-EGFR therapy.

Putative high-level amplifications of *NRAS* were observed in <1% of cases in TCGA dataset though its significance in CRC remains poorly described [5]. *KRAS* amplifications have been associated with acquired resistance to EGFR inhibitors cetuximab or panitumumab in CRC preclinical models [11]. To our knowledge, we are the first to report a novel *KRAS*^R68S1^ alteration that was associated with a particularly aggressive phenotype and PD at 2 months on anti-EGFR therapy with cetuximab.

The majority of *RAF* mutations found in our population were *BRAF*^V600E^ activating mutations (exon 15), which have been historically associated with poorer survival, resistance to chemotherapy, and lack of clinical benefit with anti-EGFR therapy in mCRC [12–15]. We also identified a lone *BRAF*^L597R^alteration (exon 15), which is poorly described in CRC but has been shown to similarly activate RAF-MEK-ERK signaling in melanoma *in vitro* [16]. Of note, this patient received 9 months of first-line anti-EGFR therapy though our sample size of 1 precludes any meaningful generalizations. One *ARAF*^S214F^ alteration was also identified in a patient whose course has been characterized by multiple recurrences of rectal cancer. Mutations in *ARAF* have been linked as oncogenic drivers in lung adenocarcinoma, and are exceedingly rare in CRC and comprise approximately 2% of cases in the CRC dataset from TCGA [5, 17]. Treatment with the oral RAF inhibitor, sorafenib, has demonstrated prolonged response in a case of refractory non-small-cell lung cancer and rapid responses in patients with refractory histiocytic neoplasms bearing somatic *ARAF* mutations [17, 18].

Despite a previous conception that *KRAS* and *BRAF* mutations are mutually exclusive, we found 1 dual *BRAF*^V600E^+*KRAS*^A164V^sub^mutated tumor that, in our case, was associated with poor prognostic features [19]. One case of concurrent *BRAF*^G466V^+*KRAS*^G12S^ mutation and one patient with a concurrent *BRAF*^G469E^+*RAF1*^S257L^ mutation were present in our cohort. *BRAF* mutants G466V and G469E have been shown to represent variants with impaired or complete loss of kinase activity *in vitro* [20, 21]. Nevertheless, it has been shown that tumorigenesis is promoted in the presence of deactivating *BRAF* mutations through oncogenic *RAS* mutation and/or CRAF (or RAF-1) signaling [21, 22]. In our study, one deactivating *BRAF*^G466V^ mutation was paired with an oncogenic *KRAS*^G12S^ mutation, and one deactivating *BRAF*^G469E^ mutation was paired with an oncogenic *RAF1*^S257L^ alteration, supporting the notion of an evolutionary adaptation in the cancer genome to overcome *BRAF* mutations with impaired function. In both cases, there were associated features of poor prognosis though the dual *BRAF*^G466V^+*KRAS*^G12S^ mutated tumor has seen disease control recently on 41 cycles of FOLFIRI and bevacizumab, which may argue for varying degrees of relative contribution from each mutation on tumor phenotype. Interestingly, one patient with deactivating *BRAF*^D594G^mutation was refractory to all lines of treatment, including anti-EGFR therapy, and ultimately died of aggressive disease. This is at odds with recent reports suggesting that *BRAF*^D594G^ mutation may be an indicator of good prognosis [23]. It is unclear whether this patient's concurrent *MET* amplification may have contributed to his overall poor prognosis and therapeutic resistance.

*ERBB2* (*HER2/neu*) amplifications were found in 5.1% of our mCRC patients with the majority in *KRAS* wild-type tumors (except for 1 with a concurrent *ERRB2*+*KRAS*^G12D^ alteration). Another FoundationOne analysis of >10,000 cases of gastrointestinal malignancies identified *HER2* amplifications and mutations in 3.0% and 4.8%, respectively, of cases from the CRC cohort [24]. Our patients with *HER2* amplified tumors appeared to have shortened clinical benefit with anti-EGFR therapy, which is consistent with the recent phase II HERACLES trial where none of the patients with *HER2* amplified, *RAS*/*BRAF* wild-type metastatic colorectal tumors had a response to anti-EGFR therapy [25]. Similar to the preponderance of left colon primary tumors in the HERACLES trial, all of our *HER2* amplified tumors were located in the rectosigmoid colon. In short, the identification of *HER2* amplifications in patients with *RAS*/*BRAF* wild-type metastatic colorectal tumors is of major significance given the clinical benefit derived from dual *HER2*-directed therapy including trastuzumab + lapatinib (HERACLES) or trastuzumab + pertuzumab (MyPathway) [25, 26].

*PIK3CA*, *PTEN*, and *AKT* mutations were identified in 18.1% (25), 10.9% (15), and 2.9% (4) of our mCRC patients, respectively. Many of these patients had metastatic tumors associated with aggressive features. In addition, 75% of *AKT* mutated tumors were located in the right colon, almost half (48.0%) of *PIK3CA* mutated tumors were right-sided colon cancers, and concurrent mutations in RAS-RAF-MAPK or PTEN-PIK3CA-AKT signaling were common. For example, 19 patients (76.0%) with *PIK3CA* mutations also had concurrent *KRAS* mutations while 5 (20.0%) and 1 (4.0%) with *PIK3CA* altered tumors also had concurrent *PTEN* and *AKT1* mutations, respectively. Mutations in mediators of the PTEN-PIK3CA-AKT signaling pathway in CRC have been associated with poorer prognosis and lack of clinical response to anti-EGFR therapy [27, 28]. For *PIK3CA* mutations, in particular, prior studies have demonstrated that exon 9 mutations had no effect while exon 20 mutations were associated with resistance to anti-EGFR therapy [29]. However, this differential effect by exon has not been supported by recent meta-analysis [30]. Given the high rate of concurrent *RAS* mutations seen with *PIK3CA* and related pathway mutations, a definitive association between resistance to EGFR inhibition and PTEN-PIK3CA-AKT pathway mutations is difficult to make. Further studies are needed to resolve this issue.

Three patients (2.2%) demonstrated *MET* amplifications associated with poor prognostic features. *MET* amplification and increased c-MET expression have also been associated with an aggressive phenotype and therapeutic resistance, particularly to MEK inhibition, in mCRC [31, 32]. Interestingly, we have observed anti-EGFR refractoriness in 2 of our patients with *MET* amplifications despite the presence of a *RAS-* wild-type phenotype and lack of activating *BRAF* mutations. This is consistent with preclinical data suggesting MET activation as a mechanism of resistance to anti-EGFR therapy [33].

We lastly identified 3 patients (2.2%) with tumors categorized in the highest number of clinically significant alterations group (17-25) that also demonstrated high TMB as per FoundationOne. TMB categories per FoundationOne testing have been validated in melanoma patients treated with PD-1 blockade [34]. Response to PD-1 inhibitors was significantly superior in patients with high TMB (>23.1 mutations/MB) compared to intermediate or low TMB (3.2-23.1 mutations/MB and <3.2 mutations/MB, respectively). Furthermore, a recent phase II study showed that patients with advanced urothelial cancer who responded to the programmed death ligand 1 (PD-L1) inhibitor atezolizumab had a significantly higher TMB (median 12.4 mutations/Mb) than non-responders (median 6.4 mutations/Mb, p < 0.0001) [35]. Two patients had MSI-H tumors while 1 hypermutant tumor was MSS and harbored a *POLE* mutation. Interestingly, 42.9% of tumors with 9-16 clinically significant alterations were located in the right colon while one-third of tumors with 17-25 total alterations were located in the right colon; tumors with <9 number of alterations were predominantly located in the left colon. In the CRC dataset from TCGA, 75% of hypermutated tumors arose from the right colon yet not all of them were MSI-H [5]. Mutations in polymerase ε or *POLE* were found among 25% of hypermutated tumors in this cohort. Mutations in *POLE* have been shown to contribute to an ultramutated yet MSS phenotype in colorectal tumors [36]. A recent NGS study confirmed that increasing mutational load correlated with MSI yet colorectal tumors with the highest mutational burden that were distinct from MSI tumors all harbored *POLE* mutations [37]. Furthermore, mismatch repair-deficiency or MSI has recently been shown to predict clinical benefit to immune checkpoint blockade with anti-PD-1 therapy in mCRC [38]. The characterization of mutational load in CRC may serve as a better indicator than MSI status in determining a hypermutant profile that could predict benefit from immunotherapy. Our findings are hypothesis generating and offer support to consider molecular analysis of tumors to determine the total number of alterations as a potential correlate to MSI and candidacy for anti-PD-1 therapy in mCRC.

Future studies of larger size and, ideally, prospective design will be helpful in corroborating associations between molecular alterations of interest described in our study and prognosis, resistance to EGFR inhibition, and/or ability to be targeted for therapy in mCRC. Comparative genomic analyses have identified a high level of concordance particularly for *RAS*, *BRAF*, and *PIK3CA* mutations between colorectal primary and metastatic tumors [39, 40]. However, other molecular alterations may differ based on the site of tumor and/or exposure to chemotherapy [41–44]. Although such mixed results are likely dependent on the specific mutation that is profiled, other factors including specimen integrity and sampling method may also contribute to heterogeneity. Indeed, further analyses are needed to describe the concordance or discordance of other mutations across tumor sites and treatment effects in mCRC, and careful consideration in design will be needed in order to account for confounding factors as described above.

In conclusion, comprehensive genomic profiling can uncover gene alterations beyond conventional *RAS* or *RAF* mutant subtypes that predict resistance to anti-EGFR therapy and in identifying potential therapeutic targets outside of NCCN standard treatments in mCRC. *ERBB2* amplified tumors commonly originate from the rectosigmoid colon, are predominantly *RAS*/*BRAF* wild-type, and may predict benefit to HER2-directed therapy. Hypermutant tumors or tumors with *POLE* mutations may predict benefit to anti-PD-1 therapy. Our findings are hypothesis generating and warrant further investigation in larger datasets and in prospective settings.

## MATERIALS AND METHODS

### Study patients and tumor samples

Patients with advanced or metastatic (stage IV) colorectal cancer treated at the Gastrointestinal Medical Oncology Clinic at City of Hope National Medical Center (Duarte, CA) between April 2013 and February 2016 were screened for this study. Eligibility criteria was limited to those who underwent expanded genomic tumor analysis by FoundationOne. There were no exclusions to tumor histology, medical comorbidities, previous treatment or lines of prior therapy, or performance status. Comprehensive genomic profiling was conducted through NGS via FoundationOne (Foundation Medicine, Inc., Cambridge, MA) with reports generated from April 2013 to February 2016. The study was approved by the Institutional Review Board (IRB).

### Next-generation sequencing

Comprehensive genomic analysis was conducted on tumor samples (formalin-fixed paraffin-embedded) retrieved from surgical resection, core needle biopsies, or excisional biopsies and delivered to Foundation Medicine, Inc. The NGS assay performed by FoundationOne has been previously described and validated [45]. The initial whole-genome shotgun library construction and hybridization-based capture of 4,557 exons from 287 cancer-related genes and 47 introns from 19 genes with frequent DNA rearrangements has since been expanded to identify genetic alterations across the coding regions of 315 cancer-related genes and introns from 28 genes commonly rearranged in solid cancers.

### Study design

Retrospective analysis of genetic mutations, amplifications, or alterations present in our cohort of 138 patients with mCRC was performed through test results provided in an integrative report available via FoundationICE (Interactive Cancer Explorer). Patient demographics including age, sex, ethnicity, site of primary, stage at diagnosis, and number of previous treatments were obtained from chart abstraction of each patient's electronic medical record (EMR). Microsatellite instability classified as stable (MSS), low (MSI-L), or high (MSI-H) were abstracted from pathology reports and response to anti-EGFR therapy, when available, was described according to Response Evaluation Criteria in Solid Tumors (RECIST) criteria and obtained from medical records [46]. The total number of clinically significant alterations for each patient was determined by tallying the sum of alterations included in the panel of clinically significant variants provided by FoundationICE reports and arbitrarily categorized into 3 groups (<9, 9-16, and > 16 total number of alterations). We defined hypermutant tumors as those in the highest number of mutations group that were also found to have high TMB as validated by FoundationOne (high >23.1 mutations/MB, intermediate 3.2-23.1 mutations/MB, and low <3.2 mutations/MB) [34].

### Statistical analyses

All statistical analyses performed were descriptive and no formal statistical hypotheses were assessed. The sample size was determined by the total number of mCRC patients with FoundationOne results available. All descriptive statistics were conducted in Excel with associated formulas and functions.
